# Graph-based extractive text summarization method for Hausa text

**DOI:** 10.1371/journal.pone.0285376

**Published:** 2023-05-09

**Authors:** Abdulkadir Abubakar Bichi, Ruhaidah Samsudin, Rohayanti Hassan, Layla Rasheed Abdallah Hasan, Abubakar Ado Rogo

**Affiliations:** 1 School of Computing, Universiti Teknologi Malaysia, Johor, Malaysia; 2 Department of Computer Science, Yusuf Maitama Sule University, Kano, Nigeria; University of Sao Paulo, BRAZIL

## Abstract

Automatic text summarization is one of the most promising solutions to the ever-growing challenges of textual data as it produces a shorter version of the original document with fewer bytes, but the same information as the original document. Despite the advancement**s** in automatic text summarization research, research involving the development of automatic text summarization methods for documents written in Hausa, a Chadic language widely spoken in West Africa by approximately 150,000,000 people as either their first or second language, is still in early stages of development. This study proposes a novel graph-based extractive single-document summarization method for Hausa text by modifying the existing PageRank algorithm using the normalized common bigrams count between adjacent sentences as the initial vertex score. The proposed method is evaluated using a primarily collected Hausa summarization evaluation dataset comprising of 113 Hausa news articles on ROUGE evaluation toolkits. The proposed approach outperformed the standard methods using the same datasets. It outperformed the TextRank method by 2.1%, LexRank by 12.3%, centroid-based method by 19.5%, and BM25 method by 17.4%.

## Introduction

Automatic text summarization (ATS) produces a shorter version of the original document, that has a smaller digital size in terms of bytes, and yet still retains the same information as the original document. This process reduces large documents to a concise representation that facilitates reading and comprehension by humans. ATS is one of the most promising solutions for the current challenge of information overload [1]. This technique is necessary because the amount of textual data increases continuously [2], which makes searching for the required information portion difficult and time-consuming. ATS has diverse applications in natural language processing (NLP) and information extraction [3], including search engines [4], news summarization [5–7], social post summarization [8,9], sentiment analysis [10,11], product reviews [12,13], and image captioning [14].

ATS is classified as either extractive or abstractive based on the generated output. Extractive summarization selects the salient and most informative sentences in the documents and rearranges them verbatim to form a summary. Lamsiyah, Mahdaouy [15] described the steps of extractive summarization as three-fold: cleaning and representation of input text, scoring of sentences according to their importance, and the sentence selection step, which involves the selection of sentences with the highest scores to form a summary. Extractive summarization is further divided based on its purpose into query-based methods, for example, the methods proposed by Mangalampati and Ponnuru [16], Van Lierde and Chow [17]; domain-specific methods, such as the methods proposed by Cao, Luo [18], Gupta, Sharaff [19] or generic methods, such as the methods proposed by Alami, Mallahi [20], Alia, Noora [21]. Based on context, extractive ATS methods can be divided into indicative, such as the methods proposed by Narayan, Cohen [22] and informative, such as the methods proposed by Vollmer, Golab [23].

Abstractive summarization creates a summary by paraphrasing and rewriting the text using different words and grammar [24] and is similar to the manual summarization process used by human experts. The process is more complex than extractive summarization as it involves the use of deep language features and complex NLP processes [25–28]. ATS can be further classified based on the number of input documents into single-document summarization (SDS) and multi-document summarization (MDS). The primary difference between the two is that SDS generates a separate summary for each individual input document, whereas MDS generates a summary for many related documents [2,29].

ATS can be classified into supervised and unsupervised learning methods [24]. Supervised learning methods are broadly classified into machine learning-based methods, such as those proposed by Agrima, Mounir [30], Chen, Zhu [31]. Deep learning-based methods include the methods proposed by Narayan, Cohen [22], Alquliti and Ghani [32], Nallapati, Zhai [33], Garmastewira and Khodra [34], Tomer and Kumar [35], amongst others. Unsupervised learning methods include cluster-based methods, such as the methods proposed by Alami, Meknassi [36], Sapkota, Alsadoon [37]; graph-based methods, such as the methods proposed by Altmami and Menai [38], Uçkan and Karcı [39]; and ontology-based methods, such as the methods proposed by MacAvaney, Sotudeh [40], Yongkiatpanich and Wichadakul [41].

Graph-based methods are based on mathematical graph theory and represent text using graph structures. Typically, the model represents text sentences with graph vertices, and the relations between sentences are represented with graph edges. The graph method was first applied for extractive summarization two decades ago [42]. Erkan and Radev [43] used a graph-based method for MDS using eigenvector centrality to determine the ranks of sentences. Canhasi [44] proposed a graph-based MDS model based on a five-layered heterogeneous graph, and the similarity between sentences was calculated using universal paraphrastic embeddings. The graph-based method proposed by Moradi, Dashti [45] scores sentences by identifying the graph central nodes. El-Kassas, Salama [46] proposed a method called EdgeSumm that combines graph centrality with other techniques for automatic text summarization. Gong, Zhu [47] proposed a sentence centrality model based on directed graphs that reflects the sentence position in a document to enhance coherency. Kumar, Srinathan [48] proposed a graph-based method using the concept of a mapping scheme and closeness centrality to determine the importance of information and co-occurring patterns of words in a topic. A multilayer-based method was proposed for MDS by Tohalino and Amancio [49], which used the concept of interlayer for connecting sentences from different documents and intralayers for connecting sentences in the same document. De La Peña Sarracén and Rosso [50] used a measure of graph betweenness centrality for extractive summarization.

The concept of a hypergraph was used by Wang, Wei [50], Wang, Li [51] for query-focused ATS. Similarly. Wan and Yang [52] used the concept of an affinity graph for MDS by utilizing both inter- and intra-document diversity to determine the similarity between sentences, whilst Wang, Liu [53] applied a random walk algorithm to an affinity graph for MDS to impose diversity. Similarly, AlZahir, Fatima [54] used a multigraph model to represent text for extractive summarization and Ullah and Al Islam [55] proposed a semantic graph-based model for extractive text summarization by first extracting the predicate argument structure (PAS) of sentences that used to measure the semantic similarity between sentences.

Graph-based text summarization methods have been proposed for different languages: Arabic text [56–58], Serbian [59], Bengali [60], Malayalam [61], Indonesian [34], Chinese [62] and Ambaric [63]. However, despite the advancement**s** in ATS research, studies involving the development of ATS methods for documents written in Hausa, a Chadic language widely spoken in West Africa by approximately 150,000,000 people as either their first or second language, is still in the early stages of development. Hausa is widely spoken in Northern Nigeria, the Southern Niger Republic, and some parts of Cameroun and Ghana, among others. A graph-based ATS method has not been used in the Hausa language and the only method proposed for Hausa ATS, to date [64], is a machine-learning-based approach using the Naïve-Bayes classifier, which was trained and tested using only ten Hausa news articles. In this study, a graph-based ATS method is proposed for Hausa text extractive SDS by modifying the existing PageRank algorithm using normalized common bigram counts between sentences as initial vertex scores. The proposed method uses an undirected weighted graph model for textual representation. The text sentences are represented as graph vertices, and the edges between the nodes are determined by the similarity between the text sentences that are measured using cosine similarity.

The remainder of this paper is organized as follows. Section 2 discusses the materials and methods. Section 3 describes the proposed method in detail. Section 4 describes the dataset and details the experimental results. Section 4 presents the discussion, and Section 5 presents the conclusions and future work direction.

## Materials and methods

**Fig 1:**
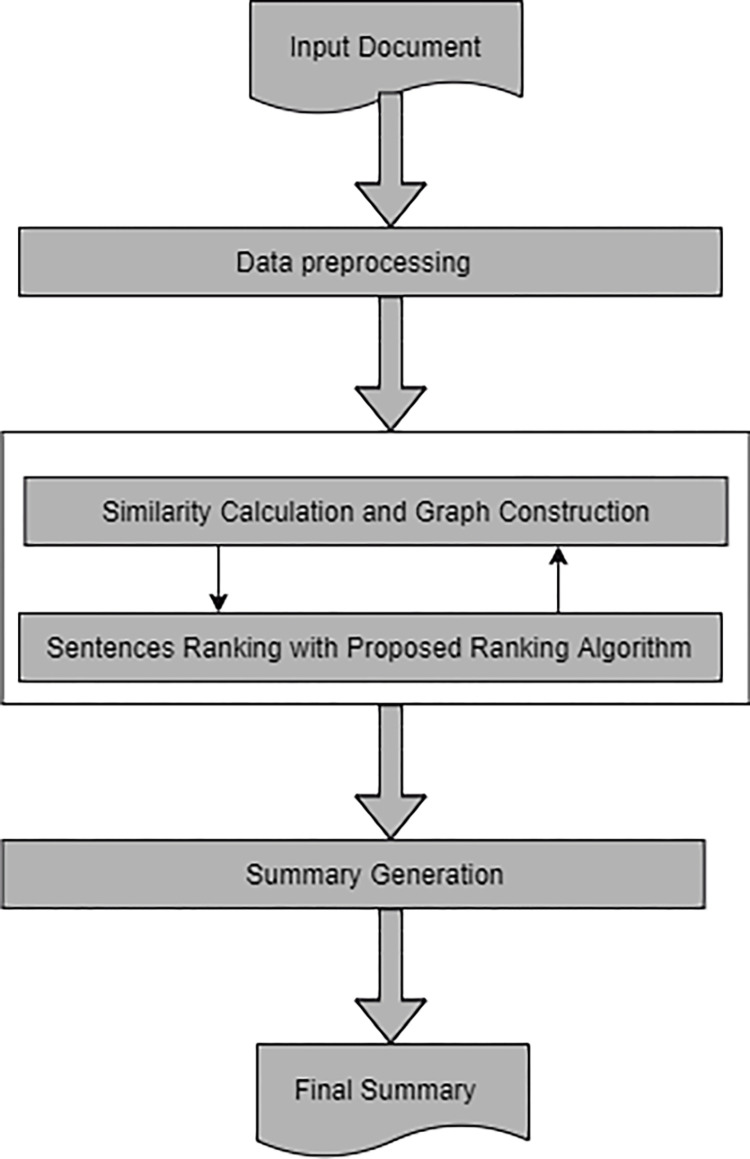
System architecture.

The proposed graph-based ATS method for Hausa text comprises four main phases: text preprocessing, similarity calculation and graph construction, sentence ranking, and sentence selection, as illustrated in Fig 1. The input of the system is raw Hausa text, and the system preprocesses the text to clean and prepare it for the subsequent stages. Subsequently, an undirected weighted graph is constructed for the text. Text sentences are represented as graph vertices, and the edges between vertices are determined by the similarity between text sentences and the proposed ranking algorithm is applied to the graph to determine the final rank of the graph vertices.

### Text preprocessing

The input text is a natural language that is unstructured and must therefore be transformed into a structured format. The preprocessing starts with case folding to convert all letters of the documents into lowercase letters and then further segmenting them into individual sentences; these are subsequently tokenized into a collection of words without punctuations. The Python NLTK library is used for both document segmentation and sentence tokenization. In Hausa text, similar to the English text, the sentences are identified with a period “.” or colon “:” marking their end, and the words are identified by a space separating them. A Hausa stemmer [65] was used to normalize words to their stem form and stop words were removed for better scoring accuracy. A list of Hausa stop words [66] was used in this study, and punctuation, non-letters, and other special characters were removed from the input text documents. We consider the following Hausa sentence: “Abubakar ya na karatu a Jamiar UTM.” The sentence is tokenized as follows: “Abubakar,” “ya,” “na,” “karatu,” “a,” “Jamiar,” and “UTM” using a space as a separator between tokens. The words “ya,” “na,” and “a” are stop-words according to the list [66], leaving only “Abubakar,” “karatu,” “Jamiar,” and “UTM” and the word “Jamiar” is stemmed to Jamia, according to the stemmer [65].

### Vector representation and graph construction

The processed text is represented as vectors of words using the term frequency (TF)–inverse document frequency (IDF) model. The text is modelled as a set D, where D = {s_1_, s_2_, …, s_n_}, s_i_ is the corresponding i-th sentence in the document and n is the number of sentences contained in D. Each sentence of the document s_i_ is represented as a vector of weights, si = (w_i_^1^, w_i_^2^, …, w_i_^m^), i = 1, 2, …, n, where wi^k^ is the weight of the term t^k^ in the sentence s_i_. In the field of information systems, there are different approaches to weighting schemes; however, term-weighting schemes have been described as the most widely used representations for extractive summarization approaches [67]. The inner product of any two sentences (represented as vectors) provides the similarity between them, as shown in Eq 1.

$$sim(x,y)=x^{T}y=\sum_{i=1}^{M}x_{i}y_{i}$$ (1)

where M is an integer representing the dimensions of space. The inner product is normalized by dividing it by the product of the vector lengths to obtain the cosine distance between them as follows:

$$cos(x,y)=\frac{x^{T}y}{\left|x||y\right|}.$$ (2)

The dimensions of the vector space are equivalent to the number of terms in the document. The term frequency (TF) is computed as follows:

$$TF(t,d)=\left\{ \begin{array}{l} 1+log(f_{t,d})\;if\;f_{t,d}>0\\ 0\;otherwise \end{array}.\right.$$ (3)

TFs are multiplied by the inverse document frequency (IDF) to overcome the challenge of domain words. IDF is expressed as follows:

$$IDF(t)=log\frac{N}{n(t)},$$ (4)

where *N* represents the total number of sentences in the document and *n(t)* is the total number of sentences containing term *t*. A constant of value 1 was added to achieve a more even result as follows:

$$IDF(t)=log(1+\frac{N}{n(t)}).$$ (5)

The products of TF and IDF are denoted as TF–IDF, and the model is known as the bag-of-words (BoW) model.

The text sentences represented as graph vertices and the adjacency matrix formed from the cosine similarities of the sentences are used to draw the edges of the graph. A similarity measure is used to determine the weights of the edges such that the weights are proportional to the strength of the causality measures between sentences. The presence or absence of an edge is determined by the value of the weights in the adjacency matrix. The edge between two sentences is considered if their adjacency value is at least 0.5, as used by Mihalcea and Tarau [42].

### Proposed ranking algorithm

This paper presents a modified PageRank algorithm for ranking sentences in Hausa text for extractive ATS. The PageRank algorithm is a ranking algorithm originally proposed for webpage analysis and is conceptualized by the premise that the importance of a webpage is determined by the number and relative importance of pages linked to it. The pages are modelled as directed graphs, and the page ranks are represented by a column stochastic matrix. The ranks are then calculated iteratively by considering the ranks of the new incoming links.

Let A denote the column stochastic matrix and v_i_ denote a vector representing the ranks at each iteration; the rank vector v is saturated at a certain value v*, known as the PageRank vector. Based on the algebraic theorem, v* is an eigenvector whose entries yield a value of 1 upon their summation. The rank of a node corresponds to the probability distribution of a random walker visiting the node. Hence, the unique vector v* in which the sequence converges is the stationary distribution value of the sequence.

The ranking problem is a graph random walk problem, which is a typical Markov chain transition problem. Similar to the Markov chain transition, an extreme condition occurs where a node known as a dangling node, which contains no outbound link, can be achieved. The original PageRank algorithm assigns a constant value of 1/n to a dangling vertex, where n represents the total number of nodes in the graph. Hence, the transition matrix of the PageRank algorithm can be defined as:

M=(1−d).A+d.B, (6)

and

$$B=\frac{1}{n}[\begin{array}{ccc} 1 & \cdots & 1\\ \vdots & \ddots & \vdots \\ 1 & \cdots & 1 \end{array}].$$ (7)

Here, *d* is the probability of discontinuing browsing the page.

In addition to the frequent occurrence of words, the modified algorithm prioritizes phrase repetition such that sentences with typical phrases have a higher probability of being selected in the summary. In this regard, a normalized bigram count common to adjacent sentences is used as the initial vertex score. A bigram is then used to estimate the probability of occurrence of a word based on the preceding word, which is calculated as follows:

$$P(W_{n}{|W}_{n-1})=\frac{P(W_{n-1}{,W}_{n})}{P(W_{n-1})},$$ (8)

where W_n_ is the word considered and W_n-1_ is the word preceding W_n_. The concept of bigrams has been applied in various NLP tasks, such as speech recognition [68] and grammar suggestions [69]. Unigram models, such as the BoW model, disregard the word order and context, and are expressed as follows:

$${W_{i}|}_{}W_{i-1}\approx W_{i}.$$ (9)

The count of typical bigrams between sentences are calculated as follows:

∅=C(bk):bk∈Si∧wk∈Sj. (10)

Eq 10 is normalized by the total count of bigrams in the two sentences.


$$\emptyset =\frac{C(bk):\;bk\in Si\wedge wk\in Sj}{C(b(Si))+C(b(Sj))}.$$ (11)

Using Laplace smoothing, a constant value of 1 is added to the numerator in the equation to avoid a zero count of bigrams.


$$\emptyset =\frac{1+C(bk):\;bk\in Si\wedge wk\in Sj}{C(b(Si))+C(b(Sj))}.$$ (12)

The original PageRank algorithm can then be modified as follows:

$$PR(uij)=\left\{ \begin{array}{l} \sum_{v\in Bu}\frac{PR(v)}{L(v)}\;if\;PR\neq 0\\ \;\emptyset \;otherwise \end{array}\right..$$ (13)

Applying a damping factor to the equation yields

$$PR(Vi)=\emptyset \;(1-d)+d*\sum_{Vj\in In(Vi)}\frac{PR(Vj)}{\left|Out(Vj)\right|}.$$ (14)

Applying the weights of the graph edges yields

$$PR^{W}(Vi)=\emptyset (1-d)+d*\sum_{Vj\in In(Vi)}wji\frac{PR^{W}(Vj)}{\sum_{Vk\in Out(Vj)}wkj}.$$ (15)

Subsequently, Eq 15 can be rewritten as

$$HS(Vi)=\emptyset (1-d)+d*\sum_{Vj\in In(Vi)}\frac{wji}{\sum_{Vk\in Out(Vj)}wkj}HS(Vj).$$ (16)

The ranking algorithm recursively computes the rank of a vertex in terms of its adjacency vertices. Given that the matrix is a column stochastic matrix, based on the Perron–Frobenius theorem, the dominant eigenvalue is 1. Subsequently, based on the power method convergence theorem, matrix converges to N, where N is the total number of graph vertices. Convergence is achieved in fewer iterations when the size of the sentences in a document is considered. Based on Langville and Meyer’s theorem, the iteration process has a time complexity of O(n^m^). The overall process of the proposed algorithm is summarized in Table 1.

**Table 1:** Graph–based Hausa text–extractive ATS algorithm.

**Algorithm: Graph-based Hausa Text Extractive ATS Algorithm**
1.Record the original sentences indices; 2.Remove punctuations and other special characters; 3.Tokenize the text into individual sentences; 4.Perform words level tokenization to further split sentences into words; 5.Normalize words to lower case; 6.Stem the individual words to their root form (using *hausastemmer*); 7.Compute the vectors representation of the individual sentences; 8.Compute cosine similarities between the sentence vectors; 9.Build text graph from the similarity matrix; 10.Compute the final rank using the proposed ranking algorithm; 11.Sort sentence in order of their ranks; 12.Select the top n sentences; 13.Rearrange the selected sentence according to their original indices

### Summary generation

The document sentences were sorted in descending order of their scores, and sentences with the highest ranks were selected and rearranged according to their original indexes in the document. The number of sentences in the final summary (FN) was determined using the assigned summary compression ratio, which was calculated using Eq 17:

$$FN=CR\times |D_{S}|$$ (17)

where CR is the compression ratio and |D_S_| is the total number of sentences in the original input document.

## Results and discussion

This section presents the corpus used for the experiment, the detailed experiments conducted, and the results obtained from the experiments. The section also presents performance evaluations to compare the performance of the proposed method with some standard methods and a detailed discussion and analysis of the experimental results.

### Dataset

Table 2 describes the details of the Hausa text-extractive ATS evaluation dataset used in this study. The dataset comprises 113 Hausa news articles from different genres, including sports, religion, politics, and culture. For each news article, there are two corresponding, manually generated gold standard summaries, whose lengths are 20% of the original article.

**Table 2:** Description of dataset.

Source	Number of articles	Total words	Number of manual summaries	Summary length	Average number of sentences in the article	Average number of sentences in the summaries
**Aminiyya newspaper**	25	7859	50	20%	10	2
**Hausa leadership newspaper**	21	9043	42	20%	11	2
**BBC Hausa**	23	6314	46	20%	9	2
**RFI Hausa**	23	6800	46	20%	5	1
**VOA Hausa**	21	6509	42	20%	11	2

### Evaluation metrics

The Recall-Oriented Understudy for Gisting Evaluation (ROUGE) [70], a recall n-gram content-based summary measure, was used to evaluate the proposed method. ROUGE supports the comparison of system summaries with more than one reference summary; it was the first proposed automatic summary evaluation tool and remains the most commonly used one [71]. ROUGE uses two metrics for the evaluation of system-created summaries: precision and recall. Precision (P) is the ratio of the number of true positives to the sum of true positives and false positives, and is defined as follows:

$$Precision=\frac{True\;positives}{False\;positive+True\;positives}.$$ (18)

Recall (R) is the ratio of sentences present in both system-generated and reference summaries to the number of sentences in the reference summary, and is defined as follows:

$$Recall=\frac{True\;positives}{True\;positives+False\;negative}.$$ (19)

The harmonic average of recall and precision is called the F-score, and is calculated as in Eq 3.


$$F-Score=\frac{2PR}{P+R}.$$ (20)

Three variants of the ROUGE simulator—ROUGE-1, ROUGE-2, and ROUGE-L—were used in this study. The ROUGE-1 metric compares the similarity of unigrams between the system-generated and reference summaries. The ROUGE-2 metric compares the similarity of the bigrams between the system-generated and reference summaries. ROUGE-L stands for ROUGE longest common subsequence, which uses the LCS metric to compare the system-generated and reference summaries.

### Experiment

To evaluate the performance of the proposed model, different experiments were conducted with 100, 200, 300, 400, and 500 iterations, as listed in Table 3. The system-generated summaries were compared with gold standard summaries using the ROUGE simulator; for each metric, the average values of the recall, precision, and F-score were recorded separately.

**Table 3:** Evaluation results for various numbers of iterations.

Metric		No. of Iterations				
		100	200	300	400	500
**Rouge-1**	Recall	64.7000	65.3000	67.6000	70.5000	70.9000
	Precision	65.8000	68.6000	70.6200	71.7300	73.7300
	F-Score	65.2454	66.9093	69.0770	71.1097	72.2873
**Rouge-2**	Recall	33.1320	33.1972	34.7761	35.1411	35.9422
	Precision	30.1740	30.4612	31.9312	32.7760	32.9120
	F-Score	31.5839	31.7704	33.2930	33.9174	34.3604
**Rouge-L**	Recall	69.7250	69.8510	69.9610	70.1340	70.6510
	Precision	68.0150	68.7350	68.7560	69.0860	70.3119
	F-Score	68.8594	69.2885	69.3533	69.6061	70.4810

### Comparison with standard methods

The performance of the proposed method was compared with that of some selected standard extractive summarization methods on the same Hausa dataset. The following methods were selected for the performance comparison: TextRank, LexRank, centroid-based, and BM25-TextRank. The TextRank method [42] was the first graph-based method for extractive summarization based on the concept of the PageRank algorithm, which represented document sentences using the vertices of an undirected weighted graph; the edges of the graph were determined using a measure of word overlap between sentences. LexRank [43] is a graph-based method for extractive summarization that uses the concept of eigenvector centrality to determine sentence ranks. The centroid-based method [72] is an unsupervised text summarization method based on a word-embedding technique that utilizes continuous vector representation to capture the semantic meaning of words. The BM25-TextRank method [73] is a combination of TextRank and BM25 ranking function that used for ranking objects in information retrieval tasks using a probabilistic model.

**Fig 2:**
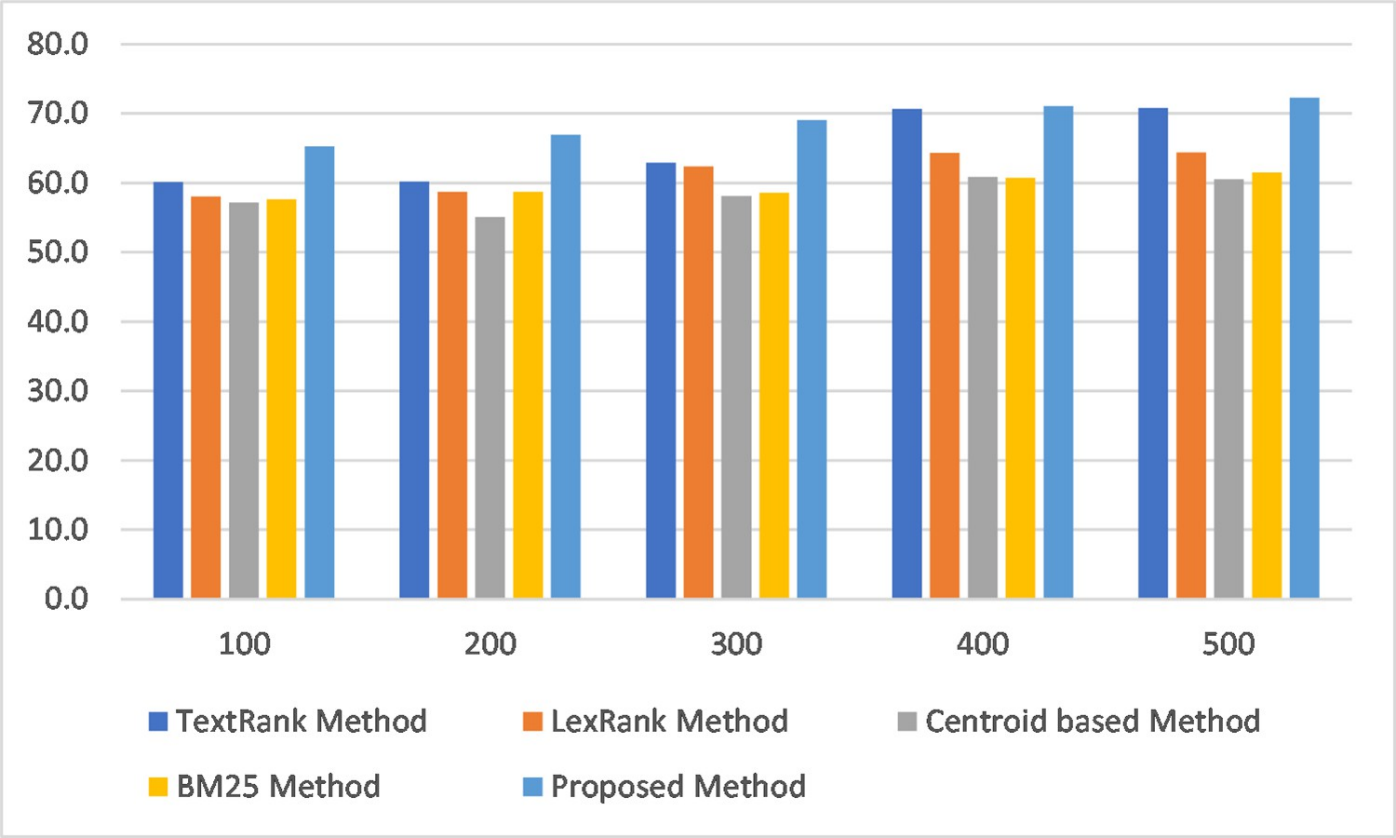
Bar chart showing experimental results.

Table 4 and Fig 2 illustrate the results of the experiments, as detailed in the Discussion section.

**Table 4:** Comparison with some standard methods.

No. of Iterations	TextRank Method (f-score)	LexRank Method (f-score)	Centroid based Method (f-score)	BM25 Method (f-score)	Proposed Method (f-score)
**100**	60.1	58.0	57.2	57.7	65.2
**200**	60.2	58.7	55.1	58.7	66.9
**300**	63.0	62.4	58.1	58.6	69.1
**400**	70.7	64.3	60.9	60.7	71.1
**500**	70.8	64.4	60.5	61.6	72.3

## Discussion

Table 4 presents the results of the experiments using the proposed method and other standard methods. The average precision, recall, and F-scores under different numbers of iterations were compared, and the proposed method outperformed all the remaining four methods using the same dataset for all metrics of Rouge-1, Rouge-2, and Rouge-L.

The experiments results showed that at 100 iterations, the proposed method outperformed the TextRank method by 8.5%, LexRank with an average F-score of 12.4%, Centroid-based method by 14.0%, and BM25-TextRank Method by 13.0%. At 200 iterations, the proposed method outperformed the TextRank method by 11.1%, LexRank method by 14.0%, Centroid-based method by 21.4%, and BM25-TextRank Method by 14.0%. At 300 iterations, the proposed method outperformed the TextRank method by 9.7%, LexRank method by 10.7%, centroid-based method by 18.9%, and BM25 method by 17.9%. At 400 iterations, the proposed method outperformed the TextRank method by 0.6%, LexRank by 10.6%, centroid-based method by 16.7%, and BM25 method by 17.1%. At 500 iterations, the proposed method outperformed the TextRank method by 2.1%, LexRank by 12.3%, centroid-based method by 19.5%, and BM25- TextRank method by 17.4%. The performance of the methods improved with an increasing number of iterations, but saturated after 500 iterations. The results obtained from the experiments and various analyses shows that the proposed method, which is an enhancement of the PageRank algorithm that uses the normalized common bigram count between adjacent sentences as the initial vertex score, outperforms the baseline methods using the same Hausa text summarization dataset.

## Conclusion

This paper presents a novel graph-based extractive single-document summarization method for Hausa texts. The method was designed by modifying the PageRank algorithm using normalized common bigram counts between adjacent sentences as the initial vertex scores. Experimental results showed that the proposed method outperformed the baseline methods using the same datasets for all metrics of Rouge-1, Rouge-2, and Rouge-L. The main contribution of this study is the introduction of a new ranking method for Hausa text-extractive summarization. The proposed unsupervised method can also be applied to any language with lexical polysemy.

In the future, the following will be explored: extending the ranking method to multi-document extractive summarization by combining it with other techniques to reduce redundancies associated with multi-document summarization. Other similarity measures should be used along with a ranking method to determine the performance of the method using different similarity measures.

## Supporting information

S1 Data (RAR)
